# Computational Phenotype Discovery Using Unsupervised Feature Learning over Noisy, Sparse, and Irregular Clinical Data

**DOI:** 10.1371/journal.pone.0066341

**Published:** 2013-06-24

**Authors:** Thomas A. Lasko, Joshua C. Denny, Mia A. Levy

**Affiliations:** 1 Department of Biomedical Informatics, Vanderbilt University School of Medicine, Nashville, Tennessee, United States of America; 2 Department of Medicine, Vanderbilt University School of Medicine, Nashville, Tennessee, United States of America; 3 Vanderbilt-Ingram Cancer Center, Vanderbilt University School of Medicine, Nashville, Tennessee, United States of America; Children’s National Medical Center, United States of America

## Abstract

Inferring precise phenotypic patterns from population-scale clinical data is a core computational task in the development of precision, personalized medicine. The traditional approach uses supervised learning, in which an expert designates which patterns to look for (by specifying the learning task and the class labels), and where to look for them (by specifying the input variables). While appropriate for individual tasks, this approach scales poorly and misses the patterns that we don’t think to look for. Unsupervised feature learning overcomes these limitations by identifying patterns (or *features*) that collectively form a compact and expressive representation of the source data, with no need for expert input or labeled examples. Its rising popularity is driven by new *deep learning* methods, which have produced high-profile successes on difficult standardized problems of object recognition in images. Here we introduce its use for phenotype discovery in clinical data. This use is challenging because the largest source of clinical data – Electronic Medical Records – typically contains noisy, sparse, and irregularly timed observations, rendering them poor substrates for deep learning methods. Our approach couples dirty clinical data to deep learning architecture via longitudinal probability densities inferred using Gaussian process regression. From episodic, longitudinal sequences of serum uric acid measurements in 4368 individuals we produced continuous phenotypic features that suggest multiple population subtypes, and that accurately distinguished (0.97 AUC) the uric-acid signatures of gout vs. acute leukemia despite not being optimized for the task. The unsupervised features were as accurate as gold-standard features engineered by an expert with complete knowledge of the domain, the classification task, and the class labels. Our findings demonstrate the potential for achieving computational phenotype discovery at population scale. We expect such data-driven phenotypes to expose unknown disease variants and subtypes and to provide rich targets for genetic association studies.

## Introduction

One of the key advances necessary to achieve precision, personalized medicine will be to transition away from using historical, clinically driven descriptions of each disease, and instead to allow the data to speak for themselves, to tell us what all of the phenotypes really are. This perspective is supported by recent results indicating that long-recognized diseases such as asthma or heart failure are not really single entities, but instead are collections of many different phenotypes that may or may not coincide with historical disease boundaries [1]–[3].

The unbiased, data-driven *phenotype discovery* necessary for this advance could conceivably be achieved using a massive biomedical dataset and a computationally intense analysis capable of identifying all of the phenotypes in the dataset. The analysis might be aimed at identifying all of the unanticipated effects, beneficial and adverse, of any given medication; or at identifying all undiscovered subtypes of every known disease; or at identifying all clinical phenotypes that have never been seen in the past, but appear to be emerging now. These kinds of analyses require computational algorithms capable of coping with not only the massive size of the dataset, but also the massive scope of the phenotype discovery task.

Traditionally, the task of finding phenotypic patterns in biomedical data has been undertaken one specific question at a time using *supervised learning*, in which a computational algorithm searches for patterns among input variables (or *features*) that model a specific outcome variable. For example, we might build a logistic regression model to identify the phenotypes of patients at risk for developing acute kidney injury during a hospital admission [4]. In this approach a domain expert decides on the specific question or *learning task*, constructs potential features as inputs, and prepares the outcome values for training and testing the model.

While this approach has served well for decades, it has limited capacity to scale beyond individual models developed to predict or explain pre-specified outcomes. The skillful identification of input features is typically the key step in determining the accuracy of a supervised model, and it takes a great deal of effort. The preparation of the outcome variable (which is often a binary *class label*) can also be labor intensive, commonly requiring an expert to examine every last input instance and designate its outcome label. These manual steps limit the speed with which we can develop a supervised model.

A much greater limitation of using supervised methods for phenotype discovery is the fact that supervised methods find only the patterns that we choose to look for (by specifying a learning task and an outcome variable), and only where we choose to look for them (among the predefined features). So while supervised learning is good at finding patterns that explain phenotypes we know enough to label in advance, it is unsuited to the scenario in which we don’t know enough to label the phenotypes, and we wish to discover them from the data.

Recently, an alternative approach known as *unsupervised feature learning* has proven to be scalable in the number of input records as well as in the scope of the learning task, in part because it is capable of finding its own patterns with little human guidance [5]. These properties make it a good match to the task of phenotype discovery. Rather than relying on an outcome label to define which patterns are important, unsupervised feature learning finds a set of patterns that form a complete representation of the entire source dataset (meaning that any record in the source data can be accurately constructed as a combination of the learned patterns). This learned representation illuminates potentially unknown structure and captures characteristic variations in the landscape of data. The elements of the learned representation can serve as a complete and powerful yet general set of input features for multiple different downstream learning tasks [5]. Because of this common use, the learned patterns that form the new representation are called *features*.

Unsupervised feature learning has recently become the state of the art in certain image object detection tasks [6]; in this paper we introduce its use for clinical phenotype discovery. We also present an approach to overcome the difficulties of applying unsupervised feature learning to the noisy, sparse, and irregular data typically found in an Electronic Medical Record, and we give encouraging results from a simple demonstration project.

Previous work using unsupervised methods to find useful patterns in longitudinal biomedical data has focused on extracting those patterns from a symbolized representation of regularly and densely sampled time-series [7], [8], identifying repeating patterns among discrete events [9], aligning the times of index events and searching for common patterns in the distribution of related events [10], converting measurements to an interval-based temporal abstraction [11] and then using frequent itemset mining to find common patterns [12], [13], or ignoring time all together and using vector-space methods for feature learning [14]. Unsupervised feature learning is different because rather than finding patterns that are simply common, it finds a set of patterns that form a complete and compact (though possibly approximate) representation of the source data. Our own work builds on standard unsupervised feature learning methods by allowing continuous, longitudinal features to be learned from noisy, sparse, and irregularly sampled data, and accounting for the inherent uncertainty of doing so.

Related work that reaches back fifty years [15]–[18] investigates *supervised* feature learning, where the expert selects the learning task, a set of raw input variables and the outcome labels, and then the algorithm tries to construct features from the input variables either individually [12], [15], [16] or as an entire feature space [19] that best predict the outcome. These methods can reduce some of the human bottleneck, or they can be used where there is insufficient domain knowledge to generate manual features for a given task. But they only find features with task-specific utility, they are as a rule not as effective as features engineered by an expert for a specific task [20], and they don’t seek to provide a complete representation of the input data.

New enthusiasm for unsupervised feature learning has emerged with the discovery of *deep learning*, an approach to constructing a hierarchy of progressively complex feature layers with each layer forming a complete data representation [5], [21], [22]. (The word *deep* refers to the number of layers in the feature hierarchy.) Although deep learning operates using no domain knowledge, in some cases it produces, *all on its own*, features that resemble the products of decades of feature-engineering research. For example, deep learning produces edge and spot detectors as translation-invariant features for photographic images [23], stereotyped object parts as features for images of objects [23], and phonemes as features from speech recordings [24]. It is not too much of a stretch to claim that, at least in some areas, a deep learning algorithm constructs from a single large dataset domain knowledge rivaling that of decades of research. Unsupervised features have met or exceeded the state of the art on several standardized machine-learning tasks. Recent results using the largest deep architecture to date achieved a 70% improvement over the previous best performance on an extremely difficult object recognition task, where each object belonged to one of 22,000 different categories [6]. It would be prohibitively time consuming to train a separate supervised classifier for each category, but a single (very large) deep architecture learned features that handled all categories at once.

Our hope is that deep learning will prove to be as successful with phenotype discovery as it has been for object detection. We expect that it will produce a similarly appropriate representation of biomedical data that saves us the decades of labor often needed to design a hand-crafted, general-purpose alternative. We also expect that it will provide new insight into the structure of the data, and we hope that this new insight will illuminate new details of the nature of disease and therapeutic processes.

An obvious data source for clinical phenotype discovery research is a large-scale Electronic Medical Record (EMR). But because EMRs are primarily designed for clinical care, adapting them for secondary use as a research data source requires some effort. EMRs have previously been used in clinical [25]–[27] and genomic [28], [29] research by leveraging expert domain knowledge to manually engineer phenotype specifications that identify clinical cohorts of interest. A recent example includes cohorts of individuals with type II diabetes, rheumatoid arthritis, Crohn disease, multiple sclerosis, or atrial fibrillation and their corresponding controls [30]. These phenotype specifications are typically composed of Boolean predicates and logical operators over structured data such as laboratory values, medication doses, vital signs, billing codes and concepts extracted via natural language processing of unstructured narrative clinical text [31]. Creating these specifications can take months of labor and interaction between clinical experts and informaticians. Some progress has been made using supervised machine learning to define disease-level cohorts [32], [33], which avoids the manual phenotype specification but still includes all of the other effort associated with supervised methods. Moreover, it is a nontrivial task to compactly represent the time-dependent, longitudinal behavior of clinical variables that could help define the cohort. Our research seeks to replace these labor-intensive efforts with unsupervised methods that computationally find the phenotypic patterns, including characteristic longitudinal variations, needed for any downstream cohort definition.

Unfortunately, standard deep learning on its own cannot easily learn compact longitudinal features from the noisy, sparse, and irregular observations typically contained in an Electronic Medical Record. To bridge this gap, we introduce the use of Gaussian process regression to transform the raw data into a continuous longitudinal probability density.

In the following sections we present the details and results of a demonstration project using these methods. The experiment was the simplest we could devise that would test the potential of unsupervised feature learning for longitudinal clinical phenotype discovery from episodic EMR data. In this experiment, each learned feature represents a phenotype discovered from the set of time-dependent clinical measurements of a single variable in deidentified medical records. The features function as low-level, high-resolution phenotypes analogous to the edge detectors typically learned by the lowest architectural layers using image datasets.

Our evaluation assessed the face validity of the learned phenotypes, their ability to illuminate potentially unknown disease population subtypes, and their ability to distinguish large-scale disease phenotypes known to exist in the data, but which were unknown to the feature learning algorithm. We compared the discrimination capability of the learned features to that of features engineered by an expert and tailored to the specific discrimination task, and found the expert-engineered features to be no more powerful than the learned features, despite the considerable advantages given to the design of the expert features.

## Results

In this project we learned continuous, unsupervised features from longitudinal serum uric acid measurements made at irregular points in time, separated by intervals ranging from hours to years. Uric acid is the end product of purine metabolism in humans. It is excreted by the kidney and has a normal serum concentration of about 3.5 mg/dl in infants, gradually increasing to about 6 mg/dl in adults in developed countries [34], [35]. Elevated concentration results from increased production or decreased excretion. Sustained high concentration can result in needle-like crystals precipitating out of solution and causing substantial pathology. In the disease gout, any of a number of genetic mutations combined with environmental factors cause elevated uric acid concentration, with crystals precipitating into joints and causing an exquisitely painful arthritis [36]. Additionally, kidney stones or kidney failure can occur if the crystals precipitate in the urinary tract. In some neoplastic diseases such as acute leukemias, continuous white blood cell turnover raises uric acid concentration throughout the course of the disease, and tumor lysis syndrome [37] can occur with the bolus of purines released from cells after a chemotherapy treatment. In both gout and acute leukemias, uric acid levels are monitored and treated with medication. The different pathophysiology of the two types of diseases combined with their different treatment goals and protocols tends to give rather different longitudinal signatures to their uric acid measurements. In this project, we tested whether our learned features could differentiate the gout vs. the leukemia phenotype signature manifest in the uric acid concentration over time.

Our feature-learning approach consisted of a transformation step using Gaussian process regression followed by a feature learning step using deep learning. While both Gaussian processes and deep learning are well known in the machine learning community, we will briefly describe them below, because they are not as widely used in the biomedical domain. Our descriptions will follow the concrete, simple example of our demonstration project, but they readily generalize to other data types and greater phenotype complexity.

### Data

After obtaining IRB approval, we extracted all necessary data from Vanderbilt’s Synthetic Derivative, a deidentified mirror of our production EMR [38]. This mirror contains over 15 years of longitudinal clinical data on over 2 million individuals. For simplicity, we will use the term *sequence* to refer to the set of all time-stamped uric acid measurements in a single individual’s deidentified medical record.

We identified 4368 records of individuals with either gout or acute leukemia, but not both (Table 1). We extracted the full sequence of uric acid values and measurement times from each record and associated it with the appropriate disease label. The disease label served as the reference standard for downstream evaluation, but was not used in the feature learning. Roughly a third of the records were set aside as a final test set.

**Table 1:** Statistical characteristics of uric acid sequences in gout vs. leukemia.

Attribute	Gout	Leukemia
Number of Sequences	2194	2174
Minimum	0.9	0.0
1st Quartile	6.2	3.0
Median	7.7	4.2
3rd Quartile	9.5	5.6
Maximum	34.0	75.0

### Transformation Step

The transformation step assumed that there was an unobserved *source function* for each individual that represented the true uric acid concentration over time, and considered each uric acid sequence to be a set of possibly noisy samples taken from that source function. We like the transformation step that infers this source function from the sampled data, but this inference is an example of an ill-posed inverse problem – there are an infinite number of functions that could fit the data [39]–[41]. To make any judgments on candidate functions we must impose some kind of constraint that allows us to prefer one over another. Proposing suitable constraints is one of the few places in our method where domain knowledge and human judgement is used. As we will see, these constraints can be rather vague expressions of how we think pathophysiologic processes tend to behave.

#### Gaussian process regression

Gaussian process regression is a Bayesian nonparametric method that provides weak constraints on candidate functions without imposing parametric forms on them. The topic is a large one for which extensive tutorials are available [42]–[44]. We outline here the prior results from the Gaussian process community needed to understand our approach, and we state them in a slightly less general form that is directly applicable to our context.

The weak constraint imposed on candidate functions by Gaussian process regression is a *Gaussian process prior*, which we will briefly explain. A *Gaussian process* is a useful way to represent a probability density 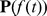 over arbitrary continuous functions 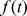. The word Gaussian in the title refers to the shape of the probability density, not the shape of any function 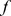. A Gaussian process can be seen as a multivariate Gaussian distribution that has been generalized to an infinite number of variables, with each variable representing a point in time. So just as we define the probability of a given vector of points 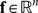 in terms of an *n*-dimensional multivariate Gaussian distribution 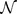 with a mean vector 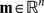 and a covariance matrix 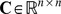, or.
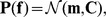
we likewise define the probability of a given continuous function 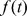 in terms of an infinite-dimensional Gaussian process 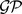 with a mean function 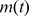 and a covariance function 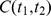, or



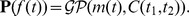



The mean function *m* is a function of time, and the covariance function *C* is a function of the pair of times 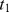 and 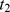. The covariance function defines the dependence between two function values 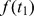 and 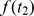. Following the common practice, we defined the mean function 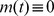, and allowed the density 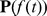 to be completely defined by the covariance function 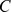.

The Gaussian process defined by 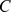 represents a prior probability density over all possible source functions for the given sequence. Gaussian process regression produces a second Gaussian process that represents a posterior probability density given the prior and the observations in the sequence.

Gaussian processes have the critically useful property that while they model the probability density of the continuous function 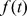, we can calculate the densities 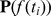 at a finite set of times 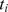, and get the same values as if we had calculated the entire 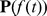 and then sampled it at the times of interest 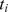. This allows the regression calculations that follow to be tractable.

Given a vector of observations 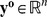 made at times 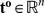, we would like to compute the posterior probability 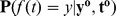 that the true source function 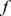 passes through the point 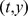. This is the same as the probability that a new measurement made at time 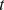 would produce the value 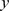.

Gaussian process regression assumes that at any time 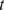, the posterior density is Gaussian, or
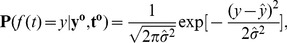
(1)where



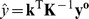
is the posterior mean value,



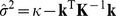
is the posterior variance, 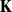 is a matrix with elements 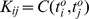, 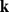 is a vector with elements 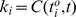, and 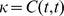 is a scalar [44], [45]. As mentioned above, this imposes a (very reasonable) parametric form only on the probability density at a given time 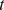, not on the shape of any potential function 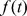.

Efficient calculation of (1) for many values of 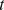 is made possible by the fact that only 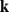 and 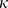 depend on the particular point 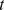. The matrix 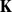 is obtained by applying the covariance function between all pairs of observed data, so it need only be calculated and inverted once (for each sequence). This inversion is the dominant step in terms of computational complexity, requiring 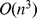 time, where 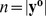 is the length of the original sequence, but most sequences are rather small, and the calculation is quite tractable.

**Figure 1:**
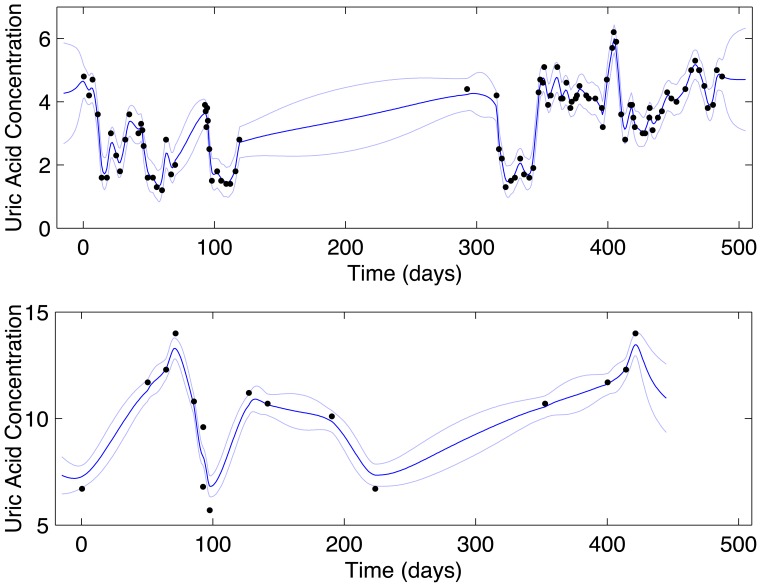
Gaussian process regression transforms noisy, irregular, and sparse observations to a longitudinal probability distribution. A cross section at any point in time in these plots is a proper Gaussian probability density centered at posterior mean 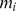 with standard deviation 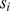. The top panel is a selected leukemia sequence, the bottom panel a selected gout sequence. Black dots: observed values. Dark blue line: posterior mean 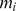. Light blue lines: standard deviation 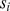.

We used (1) to compute functions representing the best estimate 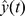, uncertainty in the estimate 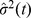, and the probability density 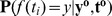 over values of 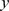, all calculated at times 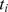. This was the goal of the transformation step. Figure 1 displays two example sequences and their estimated densities, selected from the 4592 successfully transformed sequences.

#### Covariance functions

The specifics of the estimates 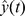 and 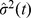 depend completely on the choice of the covariance function 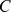, and this is where we injected our domain knowledge. In our context, we wanted 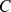 to quantify the rather simple notion that measurements made close in time should be highly correlated. The most common function used to describe this notion is the squared exponential 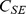:
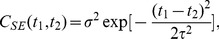
(2)where the hyperparameter 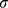 defines the magnitude of *highly correlated* and the hyperparameter 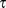 defines the time scale of *close*.

The squared exponential covariance function is often sufficient for modeling simple phenomena, but uric acid concentrations reflect a complex interaction of many different processes that may operate on several different time scales. The rational quadratic function
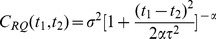
(3)is one covariance function among many that can model this complexity. It can be seen as an infinite sum of squared exponential covariance functions, each with a different time scale 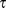 and their relative contribution defined by a gamma distribution over 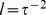, parameterized by 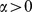 [42].

We can choose between candidate covariance functions and tune their hyperparameters for an optimum fit using several different methods [42]. We used the exact marginal likelihood of the hyperparameters (collected into the vector 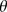) because it balances the fit against the complexity of the model:
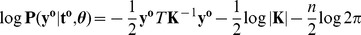
(4)


The first term assesses the fit of the observed data, the second term is a complexity penalty on 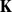 (and therefore on 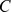), and the third term is a normalization constant. We computed (4) for each record in the dataset and optimized over the sum of all records, which produced hyperparameters 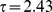, 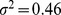, and 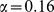. These parameters were then used to compute the regression for each sequence with (1). As an independent verification, we obtained similar results using leave-one-out cross validation within sequences, which is particularly efficient for Gaussian processes [42].

#### Time warping

Although the rational quadratic covariance function allows for a mixture of time scales, it does not allow for varying those scales as a function of time. That is, the time scale 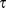 (and any other hyperparameter) is assumed not to vary as a function of 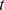. A dataset that meets this assumption is called *stationary*. Many types of clinical data, including our uric acid values, are clearly *nonstationary*. For example, if a patient is treated for acute leukemia, her uric acid level will rise dramatically, and then fall just as dramatically when she is given rasburicase to treat the hyperuricemia and prevent tumor lysis syndrome. (In practice, the drop often precedes the rise as the patients are treated in anticipation of hyperuricemia.) This can happen multiple times during a treatment cycle. A stationary Gaussian process will see these dramatic changes and determine that uric acid can change very quickly and unexpectedly, and it will inappropriately fit large uncertainties to the rest of the sequence when we would expect the values to be less volatile.

Methods exist to model this nonstationarity in Gaussian processes [46]–[48]. Rather than add their complexity to our models, however, we developed a simpler transformation that takes advantage of the tendency for clinical measurements to be made when a clinician thinks they are necessary, as opposed to a regular or a random schedule. That is, when the patient’s uric acid concentrations are more volatile due to active disease or treatment, the clinician will tend to measure them more frequently. This implies that if we shorten the longer intervals between measurements and lengthen the shorter intervals, we might bring a sequence closer to stationarity. Using domain knowledge, we constructed candidate warping functions of the form 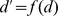, where 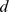 is the original distance between two adjacent observations and 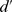 is the warped distance. We found that
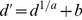
(5)provided a useful parametric form for exploring warping, and we used grid search combined with human-guided search over values of 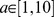 and 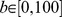 to explore possible functions. We subjectively evaluated the results based on the inferred uncertainties of the longitudinal probability densities. We selected 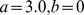 as a reasonable choice for all sequences (for example, Figure 1).

A warping approach has been previously described for spatial models [47], with the added complexity that the warping function was learned from the data. In broader use, learning the warping function may be beneficial, and would certainly be more scalable if different functions are required for different data types, but in this project we opted for our simpler method.

### Feature Learning Step

Following the transformation step, the feature learning step inferred meaningful features from the longitudinal probability densities, without using any outcome label to guide the learning. This section describes the details of this step.

#### Autoencoders

Autoencoders are frequently used as the unsupervised learning element in each layer of a deep learning architecture 21,49,50. We will describe them here using prior results from the deep learning community, and identify any modifications we made to the usual practice.

At its simplest, an autoencoder is a network of three layers – an input layer with 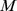 nodes representing the original data (one node per element in the data vector), a hidden layer with 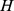 nodes representing a transformed version of the data, and an output layer with 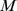 nodes representing a reconstruction of the input data using only the values in the hidden layer. The first transformation is called the *encoder*, and the second is the *decoder*.

The encoder typically transforms an input data vector 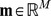 into the hidden or transformed representation 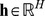 by
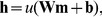
(6)where the matrix 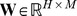 is a matrix of learned weights, the vector 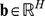 is a vector of learned bias offsets, and 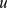 is a pre-specified nonlinear function such as the logistic sigmoid 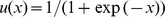.

The decoder computes a reconstruction 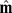 using the analogous function
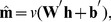
(7)where the function 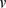 is generally either a sigmoid function if the input data values 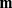 are binary, or the identity function (producing a *linear decoder*) if the input data values are continuous. The weights in 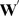 can either be learned separately or tied such that 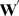 = 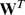.

The weights and biases are typically learned with a convex optimization algorithm such as L-BFGS [51], [52]. The cost function for this optimization is usually the squared-error loss
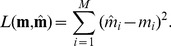
(8)


In our specific problem we wanted to reconstruct the predictive means 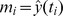 from (1) while taking into account the uncertainties 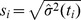, so instead of (8) we optimized the normalized squared error
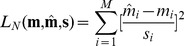
(9)that allows a looser reconstruction in areas where the uncertainty is high.

If we were to use a hidden layer larger than the input layer (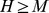), it would be trivial for the autoencoder to learn the identity transform and perfectly reproduce the input. We can avoid this trivial result by forcing dimensional reduction with 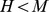, but alternatively it can be advantageous to learn an overcomplete (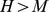) but regularized (elements of 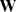 are small) and sparse (most elements of 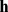 are near zero) representation. To promote regularization and sparsity we added appropriate terms to the loss function, to arrive at an overall cost function 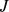:
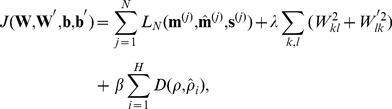
(10)where the superscript 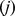 denotes the 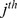 data instance (of 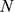 total instances), the sparsity measure 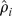 is the average activation of the 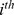 hidden node



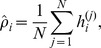
the function 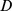 is the Kullback-Leibler divergence [53]



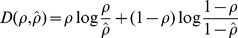
that drives all 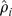 toward the sparsity target 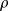, and 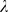 and 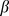 are tunable parameters to adjust the influence of the regularization and sparsity factors. This cost function produces a *sparse autoencoder* [50].

Once the weights in 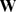 are learned, each row 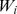 is a vector representing one of the learned features or phenotypes that are the goal of this step. (The weights 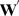 are not used beyond training.) The 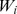 form a compact set of prototypical inputs that combine nonlinearly to form the input data vectors. If regularization and sparsity are enforced, the features 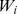 can be interpretable in the domain of interest. For example, if the original data are natural images, the lowest layer of features turns out to be a set of oriented edge and grating detectors, and higher layers consist of recognizable parts and views of objects in the input dataset [54].

Given a set of learned features 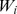, any data vector 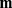, including a previously unseen one, can be represented in terms of those features using (6). The resulting element 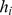 is the *activation* of feature 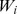 for the input 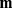, indicating how strongly 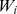 is present in 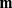.

The property of autoencoders that allows their use in a deep architecture is that they can be stacked and trained individually from the bottom up. The input instances for each new encoder are the activations 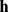 from the encoder below. Each layer learns its own weights by reconstructing its own input, and features learned by higher layers are more complex than those learned by lower layers. The progression in complexity is enabled by the nonlinear function 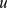 in (6), which prevents higher layers from learning simple linear combinations that could have been learned by lower layers. In this project we learned two layers of sparse autoencoders using the cost function (10), with the normalized squared error loss (9) in the first layer as written in (10), and the squared error loss (8) substituted in the second layer.

#### Learning from small patches

**Figure 2:**
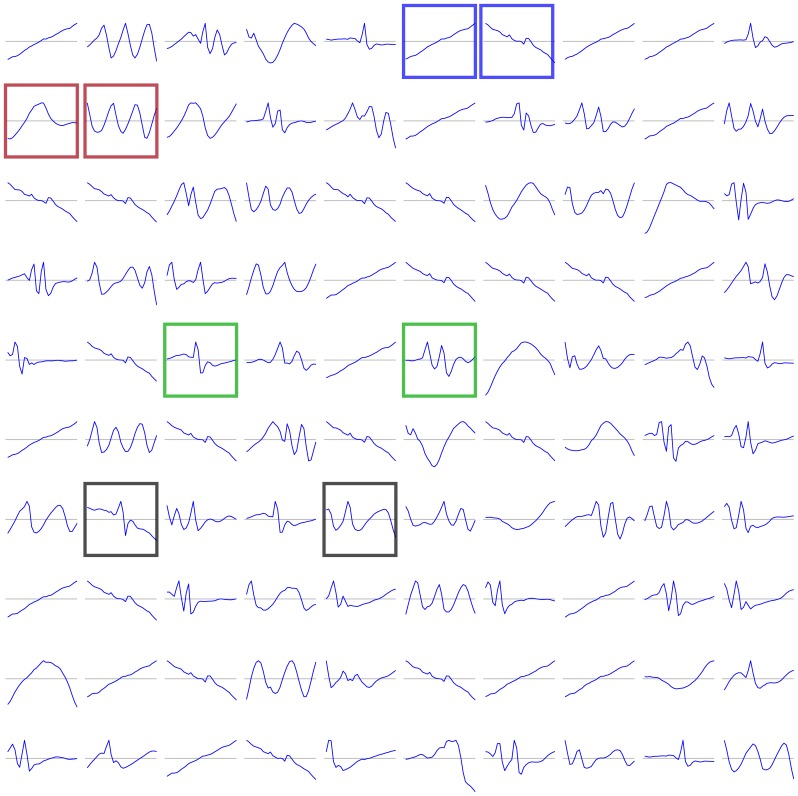
First-layer learned features are simple functional element detectors, in various combinations and phases. For example, uphill- and downhill-ramp detectors (blue), single- and multiple-spot detectors/Fourier components (red), short- and long-edge detectors (green), and mixed-element detectors (grey). These features are visualized directly as the normalized rows 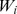.

**Figure 3:**
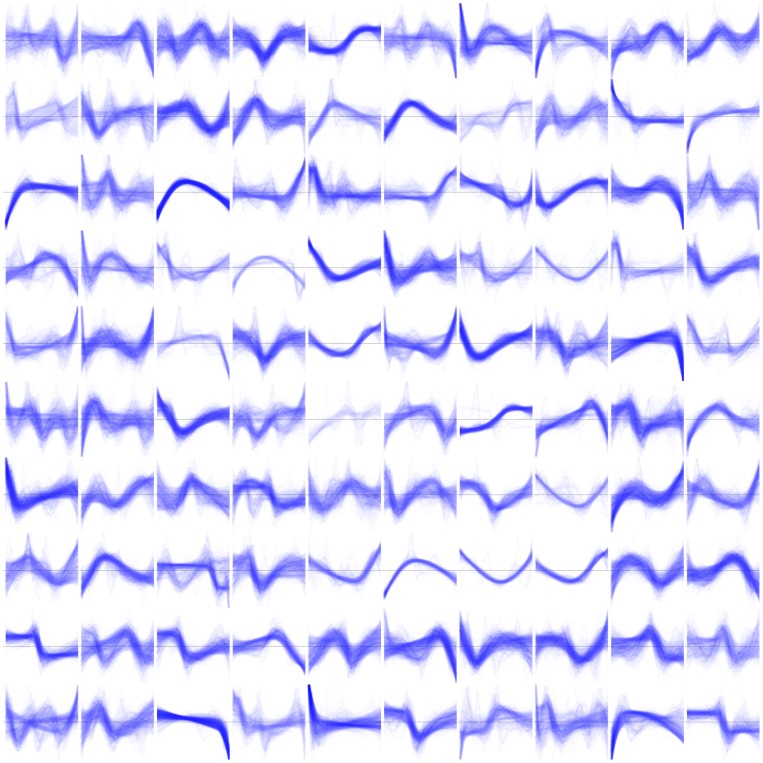
Second-layer learned features are complex nonlinear combinations of first-layer features. Because second layer features cannot be visualized directly, each feature in this set is represented as the confluence of the 100 input patches that most strongly activate the feature (those with the highest values of 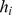 for feature 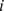).

We followed the common practice of training the autoencoders on relatively small *patches* of each input vector, rather than the whole vector at once. Specifically, we randomly extracted patches of 30-day contiguous elements from 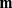 and the corresponding section of 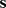, and each patch was treated as an independent training instance. Training on patches produces features that can be combined to describe 30-day trajectories of uric acid concentration (Figures 2 and 3). Training on patches improves computational efficiency, but it also has the benefit of providing features with translational invariance because a given feature can represent 30-day trajectory regardless of its position in the overall sequence.

The feature-learning step produced 100 first-layer features (Figure 2) and 100 second-layer features (Figure 3).

### Evaluation

Our evaluation assessed 1) the face validity of the learned features as low-level, high-resolution phenotypes, 2) their ability to illuminate unknown disease population subtypes, and 3) their accuracy in distinguishing between disease phenotype signatures known to exist in the data, but which were unknown to the feature learning algorithm.

On evaluation tasks 2 and 3, the performance of each layer of learned features was calibrated by comparing it to the performance of expert-engineered features. The engineered features were specifically constructed by hand to capture the essential differences in uric acid behavior in gout vs. leukemia, such as the generally lower values, more frequent measurements and wilder variations exhibited in leukemia and its treatment (Table 2).

**Table 2:** Expert Engineered Features.

Number of observations *n*
Time span of all observations *T*
Density of obervations *n*/*T*
Steepest positive slope between neighboring observations
Steepest negative slope between neighboring observations
Standard deviation of all slopes between neighboring observations
Minimum measured value
Maximum measured value
Mean measured value
Standard deviation of all measured values
Fraction of (standardized) measured values greater than 1.5

#### Face validity

The learned features are composed of continuous, interpretable 30-day trajectory components of uric acid concentration (Figures 2 and 3). The fact that the features are continuous lends face validity to the results, because nothing in the code or the constraints mandated this continuity. Interestingly, the continuity does not emerge if the regularization and sparsity constraints are absent.

The first-layer learned features represent simple functional-element detectors, in various combinations and time shifts (Figure 2). This is consistent with prior work [54], and adds further face validity. Many first-layer features are single- or multiple-edge detectors at various locations in their 30-day span. Others are single or multiple spot detectors, and some are combinations of interpretable elements. The spot detectors may function as Fourier components at periods of one-half to three cycles per patch at various phases.

Notably, there are 16 nearly identical uphill ramps and 16 nearly identical downhill ramps. These are not simply filling surplus feature slots – when we reduced the number of features from 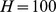 to 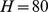, the proportion of ramps remained about the same (data not shown). The redundancy is probably an artifact of our sparsity criteria, since ramps are present in most patches, and dividing the activation between 16 features lowers the sparsity measure 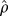. If there were only one ramp feature, its activation would be nearly saturated on every data instance.

**Figure 4:**
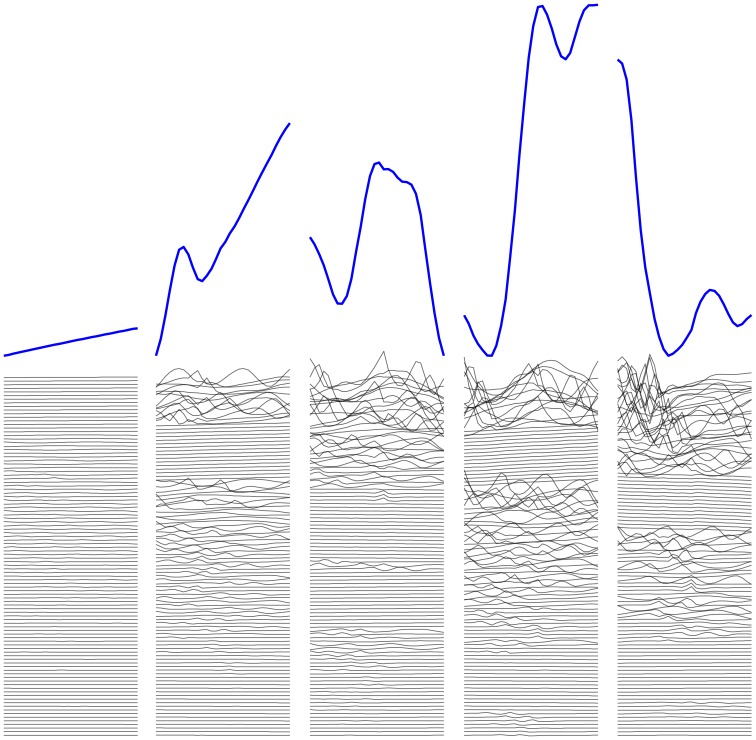
Learned features form a distributed representation and interact via constructive and destructive interference. The interference, as well as the autoencoder’s use of ramp detectors in blocks, are manifest in the confluence of features of this waterfall display. Thick blue lines: selected reconstructions of 30-day patches from the top panel in Figure 1. Stacked thin black lines: all 100 first-layer features, scaled and sorted by the magnitude of their contribution to the reconstruction.

The multiple-ramp phenomenon emphasizes that the learned features are not mutually exclusive, but rather that the information needed to represent a given input is distributed among all features acting in superposition, like simultaneous notes in a musical chord rather than sequential notes in a melody. In fact, our autoencoders learned to use constructive and destructive interference effects, where details in different features act either to reinforce or to cancel each other (Figure 4). This is important because a distributed representation can be exponentially more compact and expressive than a local representation [21].

Second-layer features were nonlinear combinations of first-layer features, and have similar face validity (Figure 3). They are slightly more complex, with a notable absence of ramp detectors.

#### Illumination of population subtypes

**Figure 5:**
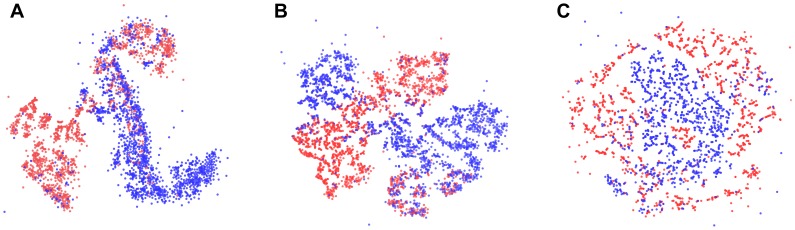
Data distribution in the learned feature spaces suggests disease subpopulations. A: First-layer features. B: Second-layer features. C: Expert engineered features. These two-dimensional embeddings using t-SNE suggest several subpopulations of gout (red) and leukemia (blue) in both learned feature spaces. We suspect that these subpopulations largely represent differences in treatment approach, but they may also be illuminating pathophysiologic differences. The engineered feature space separates the two known phenotypes adequately for a discrimination task, but offers only weak suggestions of subpopulations: without the colors corresponding to known phenotypes, it would be difficult to identify more than a single large cluster in this space. The t-SNE algorithm preserves near neighbor distances at the expense of far neighbor distances, so we cannot draw conclusions from the macro-scale shape or relative orientation of the clusters, only their number and substructure.

We investigated the landscape defined by the space of each feature set by embedding them in a two-dimensional space using t-Distributed Stochastic Neighbor Embedding (t-SNE) [55] (Figure 5). The t-SNE algorithm learns a low-dimensional embedding that preserves high-dimensional distances between near neighbors at the expense of distances between far neighbors. This tends to preserve and emphasize clusters in the original data, and any substructure within clusters. Macro-scale cluster shape and relative positions are distorted, so we cannot draw conclusions from those aspects of the embedding.

Under this visualization, all three feature sets show a separation between the two known phenotypes of gout and leukemia, but the learned feature sets (Figure 5 A and B) show additional cluster structure, suggesting subtypes among the disease populations. We conjecture that the subtypes largely reflect differences in treatment approaches, but may also reflect some underlying differences in pathophysiology. Investigating these subpopulations is a focus of future work.

The engineered feature space (Figure 5 C) arranges the data in a potentially separable way, allowing for accurate discrimination in a sufficiently flexible supervised model, but it fails to illuminate much additional structure. In fact, if the colors were removed from the figure, it would be difficult to discern more than a single large cluster.

#### Generalized discrimination performance

We objectively assessed the learned features’ ability to perform in a supervised classification task unknown to the feature-learning algorithm. This is a common method to evaluate the usefulness of unsupervised learned features [56], [57]. Because the features were not optimized for the evaluation task, the task serves as a test of generalized performance. We compared their performance to that of the expert features, which held the considerable advantage that they *were* optimized for the specific task. The selected learning task was to distinguish the known gout vs. leukemia phenotypes using only the uric acid sequences.

Our particular classification algorithm for this task was chosen to optimally illuminate differences in quality between the input feature sets. This is different from the common use of supervised learning, where the goal is to maximize predictive accuracy of the model as a whole. We chose logistic regression [58] as our classification algorithm because as a simple linear classifier it is more likely to illuminate differences in feature quality than a more sophisticated algorithm such as a support vector machine that would automatically extract complex interactions from input features to augment their descriptive power. Logistic regression is also one of the most widely used classifiers in biomedical research, so the choice also provides an evaluation of the features in a typical use case in our domain.

We trained four supervised classifiers that were identical except for the input features they used: 1) a classifier using first-layer learned features, 2) a classifier using second-layer learned features, 3) a gold-standard classifier using expert-engineered features 4) a baseline classifier using the sequence mean as the only input feature. The first two classifiers evaluated the learned features. The gold-standard classifier was intended to estimate the upper-bound performance using the best feature set we could produce for the task. The baseline classifier was intended to establish how well a single basic feature would do on the task. The gout and leukemia disease labels were used as the class labels for all classifiers.

We evaluated the performance of all classifiers using the area under the Receiver Operating Characteristic curve (AUC) [59] on a held-out test set. Despite the considerable avantage that the expert features were designed with full knowledge of the classification task and training labels, they were not able to perform better than the learned features (Table 3).

**Table 3:** Unsupervised features were as powerful as expert-engineered features in distinguishing uric acid sequences from gout vs. leukemia.

Classifier	AUC (training)	AUC [CI] (test)
First-Layer Learned Features	0.969	0.972 [0.968, 0.979]
Second-Layer Learned Features	0.965	0.972 [0.968, 0.979]
Expert Engineered Features	0.968	0.974 [0.966, 0.981]
Baseline (sequence mean only)	0.922	0.932 [0.922, 0.944]

The second column gives the performance of an Elastic Net model under cross-validation on the training set. The third column gives the performance on the held-out test set, with 95% confidence intervals determined using the bias-corrected and accelerated bootstrap. The nearly identical overlap of the confidence intervals indicates that the classifiers built from each of the two learned feature layers and the expert-engineered feature set were equally useful in the supervised learning task. Likewise, the 0.04 difference in performance between the baseline model and the other three is both statistically significant and a respectable improvement as supervised models go. AUC: Area under the Receiver Operating Characteristic curve. CI: 95% Confidence Interval.

Both learned-features classifiers performed equivalently in our logistic regression model. We believe this is partly due to the relative easiness of the supervised task (even the baseline model produced an accuracy of 0.93), and the fact that both models performed near the upper limit of what we believe is possible for this task given the input data. But it is also consistent with the goal of each layer to produce a set of expressive features that can capture characteristic signatures of the various phenotypes in the training set. Their statistically equivalent performance suggest that both layers accomplished this goal equally well, at least with respect to the gout vs.leukemia phenotypes. In higher layers, we would expect features to become more specialized and explicit, capturing more complex structure in fewer variables, and possibly progressing as far as learning a single feature for each subphenotype of gout or leukemia. In contrast, the lower layers would need to use more features in combination to distinguish the phenotypes.

All three classifiers performed better than the baseline classifier by a margin of 0.04, a respectable improvement for a supervised model (Table 3). The baseline classifier used the sequence mean as its single feature, a suitable generic baseline feature for a generic longitudinal classification task over instances with highly varying numbers of points per record. As it happened, the sequence mean was also the most predictive of all expert-engineered features, so its use here may have produced a misleadingly high baseline performance.

For all four models the test set performance was slightly better than the training set performance (but usually within the confidence interval). If not due to sampling variability, we speculate that this may be due to using cross-validation on the training set (which is convenient for the learning algorithm), but bias-corrected and accelerated bootstrap sampling (which can be more accurate) on the test set. Our primary purpose in reporting the training set accuracy is to demonstrate the absence of overfitting.

The high AUC values in Table 3 reveal the classification task to be a fairly easy one for the expert-engineered features. Indeed, we selected it because the two phenotypes appeared to have fairly distinct behavior, and we judged that a domain expert could create some effective task-specific features for the gold standard. The fact that the general-purpose, unsupervised features performed as well as the task-specific gold standard features, but without using any domain knowledge or expert input, is promising.

## Discussion

We used unsupervised feature learning for computational phenotype discovery from noisy and irregular longitudinal EMR data. Our methods achieved encouraging results in a demonstration project, in which we attempted to identify the unlabeled phenotypes expressed in the sequences of serum uric acid measurements contained in the records of people with either gout or leukemia. The learned features emerged as continuous, interpretable components of the longitudinal trajectory of serum uric acid in a given individual, which was in turn inferred as a longitudinal probability density using Gaussian process regression from discrete, episodic measurements contained in the record. Under subjective evaluation the learned features displayed considerable face validity; in an embedding analysis the features suggested unknown sub-phenotypes of both conditions; and in a supervised classification task they were highly discriminating between the two source conditions. In the classification task, the learned features performed as well as expert-engineered features, despite the engineered features’ considerable advantage of having been expressly created for the task.

To our knowledge, this is the first demonstration of deep learning over longitudinal clinical data, the first to use unsupervised feature learning for clinical phenotype discovery, and the first to use Gaussian processes to couple irregular and sparse data to a deep architecture. Our results add to a growing literature demonstrating that features produced with deep learning over large unlabeled datasets can perform as well as features engineered by an expert benefitting from knowledge of the domain, the specific learning task, and the class labels. They also add to a growing literature demonstrating that EMR data collected for clinical care can be adapted for secondary research use.

We also introduced the use of a differential time warping function that brings nonstationary clinical data sufficiently close to stationarity to allow modeling with a Gaussian process. Our choice of warping function and parameters was based on subjective expert judgment, designed to fit the particular type of nonstationarity that often arises in clinical data. Development of an objective method for optimizing the warping function is a focus of future work.

Our choice of classification task reflected our goal of automatically distinguishing populations using longitudinal characteristics of recorded data. It established that our methods are capable of capturing sufficient information to differentiate specific disease signatures in a sequence of blood chemistry measurements. We plan to attempt more demanding phenotype discovery tasks using multivariate longitudinal data in future work.

Higher levels of a deep architecture tend to produce features that are domain-recognizable sub-patterns [6], although we didn’t train enough layers to observe this in our demonstration project. With clinical data, we hypothesize that higher-level multivariate features may resemble characteristic portions of disease profiles, and may provide complex data-driven phenotypes representing disease variants and subtypes.

Our methods can be extended beyond continuous variables, to include binary or categorical variables such as billing codes, medications, narrative clinical text, or perhaps even molecular sequence information, as long as an appropriate probability density can be formulated over the observations.

We believe that computational phenotype discovery powered by unsupervised feature learning has enormous potential for advancing personalized medicine and other exciting applications. We expect high-level learned features to act as powerful data-driven, high-resolution phenotypes for applications such as syndromic surveillance, medication effects monitoring and phenome-genome association studies. Additionally, we expect that the composition of the features themselves should give new insight into the structure of biomedical data and the nature of disease and therapeutic processes.

## Methods

### Ethics Statement

The Vanderbilt University Institutional Review Board approved this research. Because the research was conducted entirely with existing data that did not include identifiable private information, the Board determined that the research does not qualify as human subjects research, in accordance with provisions of Title 45 Code of Federal Regulations part 46. Consequently, informed consent was not required.

### Data

We identified 4368 longitudinal records containing a sequence of at least two serum uric acid measurements and meeting the following criteria for either gout or leukemia (but not both). Records containing more than three ICD-9 codes for gout {274.*} and none for acute lymphoid, chronic lymphoid, acute myeloid, or chronic myeloid leukemia {204.* 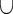 205.*} were labeled as gout records (2194 records). Records containing at least four codes for acute lymphoid or acute myeloid leukemia {204.0* 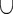 205.0*} and none for gout were labeled as leukemia records (2174 records). We extracted the full sequence of uric acid values and measurement times from each record and associated it with the appropriate disease label. All uric acid values were standardized to zero global mean and unit standard deviation.

Measurement times were date-shifted so that the first measurement in each sequence occurred on day 0, and all times were converted to fractional days. (For example, a measurement time of 6∶00 AM on day 10 was represented as 10.25).

A test set of 630 sequences from leukemia records and 634 sequences from gout records was set aside by uniform random 30% selection from the full dataset, and the remaining 3104 sequences formed the training set.

All data preprocessing was done using code we developed in R [60].

### Longitudinal Probability Density Inference

All sequences were time warped using the polynomial warping function (5) with 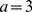 and 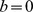, chosen as described above, using code we developed in MATLAB®.

About 20 candidate Gaussian process covariance functions of varying complexity were fit by tuning hyperparameters to the full warped training set, ignoring the class labels, using conjugate gradient optimization over the negative log marginal likelihood (4). A rational quadratic function (3) provided the best fit and was used for the rest of the work.

Posterior probability densities for each training and test sequence were estimated using Gaussian process regression at times 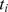 chosen at the resolution of one point per day, with 15 days of padding added before the first and after the last recorded measurement of the sequence. The daily posterior means 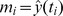 and standard deviations 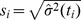 of each sequence were calculated for each day 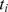 over this time range using (1). The vectors 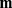 and 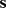 for each sequence were used as the input data for feature learning.

All density fitting used the GPML toolbox for MATLAB [61], with local additions and modifications as needed.

### Unsupervised Feature Learning

Autoencoder training instances were 30-day contiguous sub-vectors or patches extracted uniformly at random from 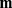 with the corresponding patch from 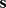. Patch extraction density was 50 patches extracted per 365 days covered by the sequence. Each patch was treated as an independent training instance, ignoring the original sequence source of each patch. The autoencoder had no access to the leukemia or gout labels.

Prior to training, each patch was standardized to give a zero mean, unit standard deviation of the mean function values 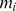. (This standardization is distinct from the global standardization prior to Gaussian process training). Specifically, we transformed the 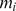 and 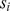 values for each patch into 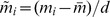, and 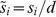, where 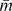 is the mean of all 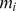 in the patch and 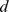 is the standard deviation of all 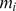 in the patch.

A two-layer stacked sparse autoencoder was trained on the set of extracted training patches. Sigmoid encoders (6) and linear decoders (7) were used for both layers. For the first layer, the number of hidden layers 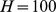, sparsity target 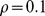, sparsity weight 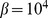, regularization weight 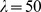 were chosen using combined human-guided and grid search, optimizing the cost function (10). Second-layer parameters 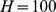, 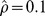, 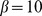, 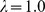 were similarly chosen, with the exception of using the squared error loss (8) instead of the normalized squared error loss (9) in the cost function (10), because the second layer used only the values 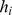 as input. Weights and biases of each layer were learned with these cost functions using the L-BFGS algorithm [51], [52].

Feature learning was performed using MATLAB starter code from Andrew Ng’s UFLDL Tutorial [50] that we substantially augmented and modified.

### Evaluation

For each model we prepared a single input instance per original sequence. Learned features were aggregated over all patches in a given sequence to produce one training or test instance per sequence. The 100 learned feature values for each patch were rescaled by multiplying by the value of 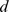 for that patch, and the maximum value of each rescaled feature over the group of patches was kept as the aggregate value for that feature. The values of 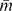 for each patch were also aggregated, keeping the mean, maximum, minimum, standard deviation, and range of the 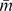 for all patches in the group. This produced 105 features for each training and test instance. The expert-engineered features are listed in Table 2. The baseline classifier used the mean of each sequence as its only feature.

#### Illumination of population subtypes

We produced a 2-dimensional embedding of each feature space (except the baseline) using t-Distributed Stochastic Neighbor Embedding (t-SNE) [55] as described in the results section. We used the MATLAB implementation developed by the authors of that method. For the visualization of both learned feature spaces, we used a perplexity parameter of 20 (which defines the length scale of interest), and for the manual feature space a perplexity parameter of 5, both chosen by human-guided optimization of the final t-SNE model score.

#### Generalized discrimination performance

We trained four models as described in the Results section, identical except for the features used as input: 1) first-layer learned features, 2) second-layer learned features, 3) expert-engineered features, and 4) the sequence mean alone.

The gout and leukemia labels were used as the class labels for all models. For each set of features we trained a logistic regression model with simultaneous feature selection via the *Elastic Net* combination of 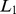 and 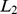 regularization [62], [63]. A regularization mixture value 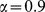 was used, weighting heavily toward 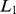, and regularization weight value 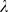 was chosen using the regularization path method [63] under 5x cross validation.

We compared the performance of each learned-features model to the engineered-features model on training and test sets using the area under the ROC curve, calculated with the nonparametric empirical method, with 95% confidence intervals calculated using 30 iterations of the bias-corrected and accelerated bootstrap [59], all using MATLAB stats toolbox implementations.
