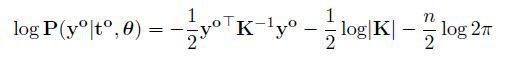# Correction: Computational Phenotype Discovery Using Unsupervised Feature Learning over Noisy, Sparse, and Irregular Clinical Data

**DOI:** 10.1371/annotation/0c88e0d5-dade-4376-8ee1-49ed4ff238e2

**Published:** 2013-08-27

**Authors:** Thomas A. Lasko, Joshua C. Denny, Mia A. Levy

There was an error in Equation 4. A correct version of the equation is available here: